# Urethroplasty is highlighted in the first number of 2023 in International Brazilian Journal of Urology

**DOI:** 10.1590/S1677-5538.IBJU.2023.01.01

**Published:** 2023-01-26

**Affiliations:** 1 Universidade do Estado do Rio de Janeiro - Uerj Unidade de Pesquisa Urogenital Rio de Janeiro RJ Brasil Unidade de Pesquisa Urogenital - Universidade do Estado do Rio de Janeiro - Uerj, Rio de Janeiro, RJ, Brasil; 2 Hospital Federal da Lagoa Rio de Janeiro RJ Brasil Serviço de Urologia, Hospital Federal da Lagoa, Rio de Janeiro, RJ, Brasil

The January-February number of Int Braz J Urol is the 20th under my supervision. In this number the Int Braz J Urol presents original contributions with a lot of interesting papers in different fields: Urethroplasty, SARS-CoV-19, Robotic Surgery, Prostate Cancer, Bladder Cancer, LUTS, Renal Cancer, Reconstructive urology and Renal stones. The papers came from many different countries such as Brazil, China, USA, Japan and UK, and as usual the editor´s comment highlights some of them. The editor in chief would like to highlight the following works:

Dr. Ma and collegues from China, presented in page 8 (1) a nice systematic review about the smoking and stricture recurrence after urethroplasty and concluded that smoking can increase stricture recurrence risk after the urethroplasty and suggested that quitting smoking may be a good option for patients undergoing urethroplasty surgery.

Dr. Tristão and collegues from Brazil, presented in page 24 (2) a interesting review about the urological complications of COVID-19 and concluded that although further studies are needed, this systematic review identified possible urological consequences or complications of COVID-19 such as changes of micturition pattern, urological urgencies, autopsies findings, sperm alterations, hormonal changes, and that the sexual transmission is highly unlikely.

Dr. Kurtzman and collegues from USA, presented in page 41 (3) the cover paper of this edition: The use of colorectal mucosa a reasonable graft alternative to buccal grafts for urethroplastaty. A very interesting study. The authors concluded that buccal grafts may continue to be a more suitable graft for urethroplasty due to their relatively thicker epithelium and thinner lamina propria, but the significant elasticity of colorectal grafts may make colorectal grafts a more suitable options in patients with longer, more complex urethral strictures, or in patients who have limited oral graft availability or oral pathology. However, the durability of this elasticity during healing remains unknown. In-vivo studies, either in animal models or humans, are needed to determine if graft selection and histologic properties affect graft take, urethroplasty outcomes and the risk of stricture recurrence.

Dr. Hakozaki and collegues from Japan performed in page 50 (4) a nice study about the predictors of urinary function recovery after laparoscopic and robotic-assisted radical prostatectomy and concluded that younger patients and patients with higher intraoperative blood loss recover urinary continence one year after surgery even if they are incontinent immediately after surgery.

Dr. Hao and collegues from China performed in page 61 (5) a study about the identification and validation of a novel prognostic model based on platinum Resistance-related genes in bladder and concluded that a prognostic model derived from platinum resistance-related genes was constructed, we confirmed its value in predicting platinum-based chemotherapy benefits and overall survival for BC patients. The model might assist in therapeutic decisions for bladder malignancy.

Dr. Riveros and collegues from USA performed in page 97 (6) an interesting study about the geriatric nutritional risk index predicts complications after nephrectomy for renal cancer and concluded that malnutrition, as defined by a Geriatric Nutritional Risk Index (GNRI), ≤ 98, is an independent predictor of 30-day complications following nephrectomy. The GNRI could be used to counsel elderly patients with renal cancer prior to nephrectomy.

Dr. Pinto and collegues from Brazil performed in page 110 (7) a validation of the Vancouver Symptom Score Questionnaire for bladder and bowel dysfunction for Brazilian children and adolescents and concluded that the translated, cross-culturally adapted, and validated VSS for the Brazilian population is a reliable and valid tool to identify symptoms of BBD in children and adolescents aged five to 16 years, whose first language is Brazilian Portuguese.

Dr. Noel and collegues from UK, Italy and USA performed in page 123 (8) a nice study about the outcomes of robotic assisted radical prostatectomy in black and white men and concluded that they could not demonstrate outcomes superiority in one group over the other. However, this data adds to the growing body of evidence that the racial disparity gap in prostate cancer outcomes can be narrowed if patients have appropriate access to prostate cancer management. It also could be used in counseling surgeons and patients on the surgical intervention and prognosis of prostate cancer in patients with full access to gold-standard screening and treatment.

Dr. Rocco and collegues from Italy performed in page 136 (9) a surgical technique showing the reproducibility of a modified posterior reconstruction during robotic intracorporeal neobladder reconfiguration and concluded that the posterior reconstruction represents a simple technical refinement that improves neobladder- -urethral anastomosis by favoring ileal approximation to the urethral stump and de- creasing anastomotic tension.

The Editor-in-chief expects everyone to enjoy reading.
